# Evaluation of the* GJB2* and *GJB6* Polymorphisms with Autosomal Recessive Nonsyndromic Hearing Loss in Iranian Population

**DOI:** 10.22038/ijorl.2020.45196.2483

**Published:** 2021-03

**Authors:** Somayeh Ebrahimkhani, Golnaz Asaadi Tehrani

**Affiliations:** 1 *Department of Genetics, Zanjan Branch, Islamic Azad University, Zanjan, Iran* *.*

**Keywords:** ARNSHL, GJB2, GJB6, Polymorphism

## Abstract

**Introduction:**

Hearing loss (HL), with more than 100 gene loci, is the most common sensorineural defects in humans. The mutations in two *GJB2* and *GJB6* (Gap Junction Protein Beta 2, 6) genes are responsible for nearly 50% of autosomal recessive nonsyndromic hearing loss. The aim of the present study was to evaluate polymorphisms of 111C>T (rs7329857) and 337G>T (rs7333214) in *GJB2* (encoding connexin 26) and *GJB6* (encoding connexin 32) genes, respectively.

**Materials and Methods:**

In this study, 32 blood samples were obtained from Iranian patients with HL defect and 32 normal blood samples were prepared. After genomic deoxyribonucleic acid extraction, genotyping in rs7333214 and rs7329857 polymorphisms was conducted using tetra-amplification refractory mutation system-polymerase chain reaction and the obtained data were analyzed.

**Results:**

In this study, the prevalence rates of CC, CT, and TT genotypes in *GJB2* gene were reported as 84.4%, 68.7%, and 0% in the affected subjects and 0%, 15.6%, and 31.3% in the control samples, respectively, which were statistically significant (P=0.004). In relation to *GJB6* gene, the prevalence rates of GG, GT, and TT genotypes were 65.2%, 78.1%, and 25% in the control subjects and 21.9%, 9.4%, and 0% in the affected samples, respectively, which were not statistically significant (P>0.05).

**Conclusion:**

The results of this study revealed that 111C>T polymorphism in *GJB2* gene was involved in the incidence of HL in the studied population and could be suggested as a prognostic factor in genetic counseling before marriage and pregnancy.

## Introduction

One per thousand neonates suffers from prelingual hearing loss (HL). Approximately every 2 to 6 in 1,000 children are affected by severe HL (1, 2). Moreover, many cases of late-onset progressive HL have a genetic origin (3, 4). The HL is a heterogeneous disorder since it could be induced by both environmental and genetic factors (5,6). Nonsyndromic HL accounts for 60-70% of inherited deafness and is induced by over 150 loci mutations. It may have autosomal recessive Deafness (DFNB; 80%), autosomal dominant (DFNA; 10%), X-linked (DFN), Y-linked (<1%) and mitochondrial (<1%) bases (7,8). 

Most of the genetic defects defined by DFNB1 locus mutations include two *GJB2* and *GJB6* genes coding Gap junction Beta-2 protein connexin 26 (Cx26) and Gap junction Beta-6 protein connexin 30 (Cx30), respectively. The Cx26 is involved in the potassium recycling of endolymph fluid; subsequently, *GJB2* mutations induce K+ recycling and lead to the necrosis of hair cells (9,10). Nearly up to 50% of prelingual recessive nonsyndromic hearing loss (NSHL) is attributed to *GJB2* mutations. More than 90 various mutations in *GJB2* gene have also been reported (11,12). However, the most common identified alleles include c.35delG, c.167delT, c.235delC, and p.R143W that these mutations affect gap-junction activity by down-regulation in numerous levels (13,14). The Cx30 is expressed in the same inner-ear structures as Cx26 and it is reported that both the connexins are functionally related to each other (15,16). The significance of *GJB6* for normal hearing has been detected by confirmation of a large deletion (GJB6-D13S1830) involving the first two exons, a part of the third exon of *GJB6* gene, and a large part of the upstream sequence (17,18). Case samples with homozygous genotype for the mentioned deletion also compound heterozygotes carrying del (GJB6-D13S1830) and deafness-causing allele variant of GJB2 have confirmed with severe to profound congenital HL (19). Several single-nucleotide polymorphisms (SNPs) have detected that covered DFNB1 locus includes rs3751385 and rs7329857 in 3' untranslated region (3' UTR), rs7994748 in intron, and rs7987302 in the downstream region of *GJB2*. Polymorphisms rs9315400, rs877098, and rs945369 located in an intron, rs7333214 in 3' UTR, and rs7322538 located in the downstream position of GJB6 gene (20,21). 

The rs7329857 (111C>T) and rs7333214 (337G>T) SNPs are located in the 3' UTR of *GJB2* and *GJB6* genes, respectively. The aforementioned mutations in this location diminished the expression of *GJB2* and *GJB6* genes and they are reported to be associated with several genetic disorders (22). Previous studies have demonstrated significant levels of HL (3 in 1,000 newborns) and consanguinity (36.8%) in the Iranian population. The purpose of the current study was to evaluate the relationship between polymorphisms in *GJB2* and *GJB6* genes and autosomal recessive nonsyndromic hearing loss (ARNSHL) in a sample of patients from the Iranian population.

## Materials and Methods

**Characteristics of clinical samples:**

The samples of this case-control study were selected from HL patients referring to genetic counseling centers in Tehran and Zanjan, Iran, during 2017-2018. A sampling of 32 deaf patients as a case group was performed in the genetic laboratory of Dr. Najmabadi-Kariminejad, genetic laboratory of Dr. Zeynli, Tehran Medical Genetics Laboratory of Dr. Akbari, Mendel Laboratory of Dr. Asaadi Tehrani, Deaf Association of Zanjan, Valiasr Hospital, and Department of Genetics in Azad University, Iran. The participants with autosomal recessive HL, suffering from moderate (40-70 decibel [dB]), severe (70-95 dB), or profound (>95 dB) NSHL and conductive HL, toxic drugs used during the pregnancy, septicemia and associated antibiotic therapies in neonates, and trauma or other environmental factors were excluded from the study. A sampling of 32 healthy individuals hospitalized in Valiasr Hospital in Zanjan was performed as a control group. It was tried to determine the correlation of the age, gender, and number in the case and normal groups. Written permission was granted from all the patients and control subjects who participated in this study by signing informed consent (medical ethics code obtained from the (Iran) IR Ethics Committee: IR.IAU.Z.REC.1397.062).

**Genomic DNA extraction:**

About 5 cc of blood was collected from each individual in special tubes containing an anticoagulant ethylenediaminetetraacetic acid (EDTA). Genomic deoxyribonucleic acid (DNA) extraction was carried out using peripheral blood samples in a volume of 500 μl containing anticoagulant EDTA of all the patients and controls by the blood genomic DNA Extraction Kit (Parstous Biotechnology, Iran) and Cat: A101201 according to the kit protocol. Subsequently, they were stored at a temperature of -80 ͦ C until usage.

**Determination of genotype for rs7329857 SNP of** **GJB2** **gene and rs7333214 SNP of** **GJB6** **gene:**

Forward and reverse primers were designed for polymorphisms, including rs7329857 of *GJB2* gene and rs7333214 of *GJB6* gene, and their manufacturing was conducted by GenFanAvaran Company in Iran (Table.1). Tetra-amplification refractory mutation system-polymerase chain reaction (PCR) was used to determine the genotype of the samples. This method was performed on the basis of the design of the allele-specific primer pairs in a PCR reaction. 

The PCR reaction was performed in a volume of 25 μl containing 100 ng genomic DNA, 12.5 μL (Master Mix) X Taq Premix2 (Parstous, Iran), 10 pM of each primer (GenFanAvaran Co., Iran) (Table.1), and 7.5 μl purified water. The PCR program consists of 32 cycles of 5 min of denaturation at a temperature of 95°C, 45 sec of denaturation at 95°C, 40 sec of annealing at 57°C for *GJB2* gene, 40 sec of annealing at 55°C for *GJB6* gene, 45 sec of extension at 72°C, and 5 min of elongation at 72°C. 

Finally, PCR products were electrophoresed on 2% agarose gel. To make sure of the determined genotypes, one sample of each genotype was amplified by outer primers and sent to Arian Gene Gostar Company in Iran for sequencing.

**Table 1:** Primers designed for Gap Junction Protein Beta 2, 6 (*GJB2* and *GJB6*) genes

**Gene**	**Primer and Sequence (5'-3')**	**Melting Temperature**(**°C) **	**Product size (bp)**
*GJB2* (FO)	CAC GGA GAA GAC TGT CTT CAC AGT GTT C	57	393
*GJB2* (RO)	AGC CTG GGG TCT CAG TGG AAC TAA CTT A	57	
*GJB2* (FI)	GCT AGC ATT TCC CAA CAC AAA GAT TCT TAC	57	264
*GJB2* (RI)	AGG GGT TTC AAA TGG TTG CAT TTA CGA	57	186
*GJB6* (FO)	GTA GCC TGA AGA GTT TGT AAA TGA CTT T	55	430
*GJB6* (RO)	TTT ACA AGA TAG ACC CTT TGT AAG TTC C	55	
*GJB6* (FI)	GGA ACA TTT ATC CAG GAA TTG ATG CT	55	284
*GJB6 *(RI)	AGC CTA TAA AAA TAT CTT TTC CTA ATA GAC	55	202

*Statistical analysis:*

SPSS software (version 19) was used to compare the differences between the blood samples of the patients with HL and healthy individuals. In order to assess the differences between the studied groups in terms of the study parameters in genotypic and allelic frequencies, the odds ratio (OR) and 95% confidence interval (CI) were utilized and considered statistically significant (P<0.05) using the Chi-square test (odds ratio calculator). 

## Results

**Fig 1:**
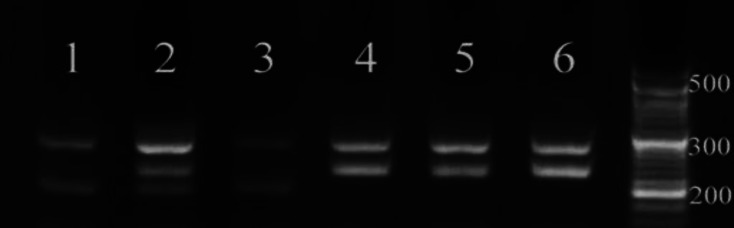
Result of polymerase chain reaction (PCR) of Gap Junction Protein Beta 6(GJB6*)* gene

In this study, the patients were within the age ranges of 32-34 and 19-88 years in the case and healthy groups, respectively. The number of individuals in the case group was 32 (16 females [50%] and 16 males [50%]). In addition, the mean ages of males and females and average age in the case group was 47, 37 and 23 years respectively. The number of individuals in the control group was 32 (18 females [56.25%] and 14 males [43.75%]). Furthermore, the mean ages of males and females and average age were 55 and 44 and 49 years, respectively. The control region for *GJB6* gene was 430 bp. Moreover, G and T alleles amplified 284 and 202 bp fragments. The control region for *GJB2* gene was 393 bp. Additionally, T and C alleles amplified 264 and 186 bp fragments. The genotypes in *GJB2* gene included CC homozygote/healthy, CT heterozygote, and TT homozygote/mutants. The genotypes in *GJB6* gene included GG homozygote/healthy, GT heterozygote, and TT homozygote/mutant (Fig. 1).

*GJB6 *genotype: GG/GT/TT TT: 1, 3 GT: 2 GG: 4, 5and 6

50 bp ladder on the right side of the gel and the PCR product within the range of 200-300 bp

**Genotypic evaluation of** **GJB2** **gene polymorphism: Total genotypic frequency**

**Fig 2:**
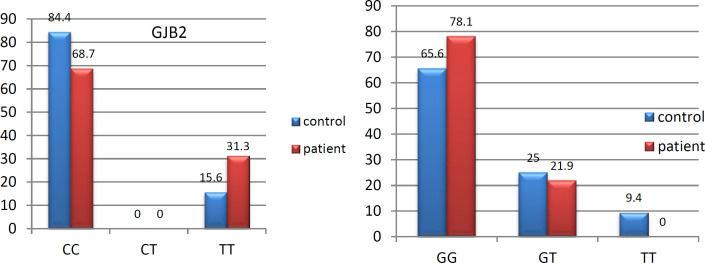
Genotypic prevalence rates of rs7329857 and rs7333214 polymorphisms in Gap Junction Protein Beta 2, 6 (*GJB2* and *GJB6*) genes in the control and case groups

In *GJB2* gene with rs7329857, the prevalence rates of CC, TT, and CT genotypes in the control group were 84.4%, 15.6%, and 0%, respectively. In the patient group, the prevalence rates of CC, TT, and CT genotypes were 68.7%, 31.3%, and 0%, respectively. The total prevalence rates of GJB2 gene were 0.76% and 0.23% for the C and T alleles, respectively (Fig.2).

In *GJB2* gene, the numbers of samples with CC, CT, and TT genotypes in the females of the control group were 12 (43.75%), 0 (0%), and 4 (12.5%), respectively. In addition, the number of samples with CC, CT, and TT genotypes in the males of the control group were 13 (40.62%), 0 (0%), and 1 (3.12%), respectively. In *GJB2* gene, the numbers of females in the patient group with CC, CT, and TT genotypes were 12 (37.5%), 0 (0%), and 4 (12.5%), respectively. Furthermore, the numbers of males in the patient group with CC, CT, and TT genotypes were 10 (31.25%), 0 (0%), and 6 (18.75%), respectively (Table 2). For GJB2 genotyping in case and control groups, the age range of females with CC, CT, and TT genotypes in this group was 21-28 years,. The age range of males with CC, CT, and TT genotypes in this group was 22 - 24 years, (Table.2).

*Evaluation of GJB6 gene polymorphism*

*Total genotypic frequency*

In *GJB2* gene with rs7333214, the prevalence rates of GG, GT, and TT genotypes in the control group were reported as 65.6%, 25%, and 4.9%, respectively. In the patient group, the prevalence rates of GG, GT, and TT genotypes were reported as 78.1%, 21.9%, and 0%, respectively. The total prevalence rates of *GJB2* gene were 83% and 17% for the G and T alleles, respectively (Fig.2).

*Genotypic frequency*

In *GJB6* gene, the numbers of heterozygote GT, homozygote GG, and TT mutant genotypes of females in the patient group were 2 (6.25%), 14 (43.75%) and 0 (0%) respectively. The numbers of heterozygote GT, homozygote, and TT mutant genotypes of females in the control group were 5 (15.62%), 13 (40.62%), and 0 (0%), respectively. The numbers of heterozygote GT, homozygote, and TT genotype of males in the patient group were 5 (15.62%), 11 (34.37%), and 0(0%), respectively. The numbers of heterozygote GT, homozygote, and TT genotype of males in the control group were 3 (9.3%), 8(25%), and 3 (9.37%), respectively (Table.2). For GJB6 genotyping in case and control groups the age range of females with GG, GT, and TT genotypes in this group was 38- 47 years. The age range of males with GG, GT, and TT genotypes was 57- 60 years (Table.2).

Genotype analysis based on Hardy-Weinberg Equilibrium (HWE) for rs7329857 (*GJB2*) and rs7333214 (*GJB6*) demonstrated that in the studied population, X^2^ detected 16.4 and 1.33 respectively (df=2). In conclusion, HWE was confirmed only for rs7333214, but not for rs7329857.

**Table 2:** Estimation of Genotypic Frequency, Total Genotypic Frequency, P-value, Number of Control and Patient Samples for GJB2 and GJB6 Genes

**Genotype**	**Frequency**		**Number of normal (32)**			**Number of patients(32)**			**P-value**
	**normal**	**Patient**	**Total**	**male**	**Female**	**total**	**Male**	**female**	
GJB2 T/T	5(15.6%)	10(31.3%)	5(15.62%)	1(3.12%)	4(12.5%)	10(30.80%)	6(18.75%)	4(12.5%)	0.0400
GJB2 C/T	0(0%)	0(0%)	0(0%)	0(0%)	0(0%)	0(0%)	0(0%)	0(0%)	
GJB2 C/C	27(84.4%)	22(68.7%)	27(84.37%)	13(40.62%)	14(43.75%)	22(68.75)	10(31.25%)	12(37.5%)	
Total allele frequency(C: 76% T:23% )			C: 0.85 T: 0.15			C: 0.69 T: 0.31			
GJB6 G/G	21(65.5%)	25(78.1%)	21(65.62)	8(25%)	13(40.62%)	25(78.12%)	11(34.37%)	14(43.75%)	0.1005
GJB6 G/T	8(25%)	7(21.9%)	8(24.99)	3(9.37%)	5(15.62%)	7(21.87%)	5(15.62%)	2(6.25%)	
GJB6 T/T	3(9.4%)	0(0%)	3(9.37%)	0(0%)	3(9.37%)	0(0%)	0(0%)	0(0%)	
Total allele frequency (G: 89% T:11%)			G: 0.78 T:0.22			G: 0.89 T:0.11			

## Discussion

According to the Human Gene Mutation Database, over 200 varieties have been reported in *GJB2* gene, out of which the 35del G mutation was the most common (70%). Other commonly reported mutations in Iran are R184P, W24X, delE120, R127H, 3170G>A (IVSI-G>A), and 235del (15). In the case of *GJB6*, the deletion of D13S1830 is the most prevalent mutation. The second common mutation is in Caucasians. More than half of the heterozygote patients of this variety have a large deletion in this gene such that one segment of 309 kb near *GJB6* and *GJB2* is deleted. It has not yet been clarified that *GJB2* gene mutations inherited in digenic model with D13S1830 del, or deleted fragment has the conventional regulatory factors for *GJB2* and *GJB6* genes (23). Furthermore, the frequency of deletion of *GJB6* gene differs in various populations. In addition, the delE120 mutation has been confirmed as the second most common mutation in the north and southwestern regions of Iran. Another common polymorphism in *GJB2* gene, including V153I, is effective in varying degrees of deafness due to a change in the encoding region of this mutation and its integration with other mutations in this gene or other genes (18).

In the present study, several SNPs covering the DFNB1 locus were screened. The most important reported SNPs are rs7329857 and rs7333214, which are reported as two common polymorphisms. The removal of nucleotides in *GJB2* and *GJB6* genes causes deafness. In studies performed on 32 deaf patients and 32 healthy controls for polymorphism rs7329857 of *GJB2* gene, there was no case of CT genotype in the patient and control groups. Moreover, the frequency of TT mutant genotype was twofold in the affected samples in comparison to that reported for the control group (31% vs. 15%) indicating a statistically significant relationship between the aforementioned polymorphism and HL (P=0.04) (11).Regarding polymorphism rs7333214 of *GJB6* gene, there was no significant correlation between the frequency of this polymorphism and deafness (P=0.1). In this study, homozygote patients were observed for *GJB2* mutations and heterozygote for *GJB6* mutations. However, no homozygote patients were observed for the TT homozygote genotype of *GJB6*. This finding confirms the results of previous studies, suggesting a lack of *GJB6* mutations in the Iranian population. According to the results of the current study, there was no relation between *GJB2* mutation with rs7329857 and *GJB6* mutation with rs7333214. In other words, there was no case of simultaneous mutants in both *GJB2* and *GJB6* genes (11). In the present study, the frequency of the G allele of rs7329857 (*GJB2*) detected in the whole population was 0.76 (G=0.68 in the affected and G=0.84 in healthy groups). A literature review of gene bank of the National Center for Biotechnology Information demonstrated a range of 0.73-1% for the G allele frequency in various ethnic groups. Analysis of relevant allele frequency database has been reported at extremely high frequency of G allele (20). For instance, In South and East Asia the frequency of G allele was determined G=1. However, in American, African, and European populations, with larger study sample size, it was reported that G= 0.73, 0.78. Additionally, analysis of allele frequency in reference population revealed rs7333214 in (G= 0.7, T= 0.302) and in the only performed study and published article, it was reported that (G= 0.77 and T= 0.23) (11). In correlation with the mentioned studies in our study (G= 0.84 T= 0.16). There is no other evidence from allele frequency of this variant and its association with ARNSHL. In the current study, the reasons for the selection of the aforementioned variants is that there have been no studies carried out on the Asian and Iranian populations regarding the association of rs7329857 (*GJB2*) variant and ARNSHL. Moreover, there has been no report in Asian or other populations for genotyping analysis of rs7333214 (GJB6). 

Grilou et al. (2011) investigated nine SNPs related to *GJB2* and *GJB6* genes. They reported that three SNPs (rs3751385, rs7994748, and rs7329857) related to *GJB2* gene and one SNP (rs7333214) related to *GJB6* gene showed a significant relationship with deafness (OR>1) and a high risk of ARNSHL (OR=11.7) for rs7329857. Furthermore, in Brazil, OR values of 11.7 and 0.46 were reported for *GJB2* and *GJB6* genes for the possibility of deafness, respectively, which increased in comparison to those of the control group. In a study conducted in Iran, OR values were reported as 2.45 and 0.43 for *GJB2* and *GJB6* genes, respectively; accordingly, the possibility of deafness increased, compared to that reported for the control group. Moreover, the frequency of CT heterozygote genotype for this polymorphism was 16%; nevertheless, in the present study, there was no individual with heterozygote genotype in the patient and control groups. 

In addition, Wilch et al. demonstrated that presence of the polymorphism rs7333214 G allele is accompanied by a reduction of the expression of *GJB2* and *GJB6* genes. In another study, Grilou reported that the T allele was accompanied by ARNSHL. The results of the present study was also in line with the findings of the study conducted by Wilch et al. indicating that 78% of the patients had GG genotype and none of them had TT genotype; nonetheless, this genotype was observed in 9.4% of the patients in the control group. Therefore, in the present study, the G allele can also be associated with ARNSHL. Grail also reported a genetic relationship with the polymorphism rs7333214 in 94% of patients. However, in the present study, the frequency of G and T alleles in the patient and control groups were 89% and 78% as well as 11% and 30%, respectively. In other words, the T allele in the control group was three times higher than that of the patient group. Nevertheless, there was no statistically significant relationship between rs7333214 frequencies with ARNSHL (3).

Matus et al. (2011), in a study in Portugal, reported that the noncoding regions of *GJB2* in rs7329857 in the c.1111C and c.111T were 174 and 4, respectively. In the control group, the numbers of c.111C and c.111T alleles were 181 and 1, respectively; nonetheless, no statistically significant relationship was observed between them (24). The molecular analysis of *GJB2* gene in Iraqi patients with a nonsyndromic sensorineural HL was conducted by Jouradat et al. (2016). The coding sequence adjacent to the exon 2 regions of *GJB2* gene for 63 patients with recessive hereditary deafness was performed, in which two frame changes and four wrong mutations were recognized in nine Iraqi patients. 

Polymorphism of 3' UTR, including c.111C> T (rs7329857) SNP, was also detected. In *GJB2* gene, the genotypic percentage was reported as TT: 3.17% in 63 patients. However, according to the results of the current study, in *GJB2* gene, the genotype percentage was TT: 31% in 32 patients (25). A major limitation of this study was the low number of samples. Therefore, it is suggested to carry out further studies with a larger sample size. Moreover, the present study could be conducted on other genes of deafness or other polymorphisms, including rs3751385, rs7994748, and rs7987302 of *GJB2* gene and rs7322538, rs9315400, rs877098, and rs945369 of *GJB6* gene. The results of the present study are in line with other studies demonstrating the role of *GJB2* gene in the incidence of ARNSHL among the Iranian population. In addition, in Iran, these polymorphisms have been investigated for the first time and have not been reported in any other studies. 

## Conclusion

The findings of this study showed that rs7329857 (C/T) polymorphism in *GJB2* gene is an effective polymorphism in increasing the risk of ARNSHL; however, rs7333214 (G/T) in *GJB6* gene does not demonstrate a significant relationship with the incidence of ARNSHL in the Iranian deaf population. Therefore, future studies on other racial groups may confirm the results of the present study.
